# Comparison of arterial spin labeled MRI (ASL MRI) between ADHD and control group (ages of 6–12)

**DOI:** 10.1038/s41598-024-63658-9

**Published:** 2024-06-28

**Authors:** You Bin Lim, Huijin Song, Hyunjoo Lee, Seungbee Lim, Seo Young Kwon, Jeeyoung Chun, Sujin Kim, Ceren Tosun, Kyung Seu Yoon, Chul-Ho Sohn, Bung-Nyun Kim

**Affiliations:** 1 Division of Child and Adolescent Psychiatry, Department of Psychiatry, Seoul National University College of Medicine, 101 Daehak-ro, Jongno-gu, Seoul, 03080 Republic of Korea; 2 Biomedical Research Institute, Seoul National University Hospital, Seoul, Republic of Korea; 3 Istanbul University-Cerrahpasa Medical Faculty Child and Adolescent Psychiatry, Istanbul, Turkey; 4 Department of Radiology, Seoul National University College of Medicine, 101 Daehak-ro, Jongno-gu, Seoul, 03080 Republic of Korea; 5 Department of Psychiatry, Hanyang University Hospital, Seoul, Republic of Korea

**Keywords:** Attention deficit disorder with hyperactivity, Arterial spin labeling MRI, Developmental change, Biomarkers, Medical research

## Abstract

This study utilized arterial spin labeling-magnetic resonance imaging (ASL-MRI) to explore the developmental trajectory of brain activity associated with attention deficit hyperactivity disorder (ADHD). Pulsed arterial spin labeling (ASL) data were acquired from 157 children with ADHD and 109 children in a control group, all aged 6–12 years old. Participants were categorized into the age groups of 6–7, 8–9, and 10–12, after which comparisons were performed between each age group for ASL analysis of cerebral blood flow (CBF). In total, the ADHD group exhibited significantly lower CBF in the left superior temporal gyrus and right middle frontal gyrus regions than the control group. Further analysis revealed: (1) The comparison between the ADHD group (N = 70) aged 6–7 and the age-matched control group (N = 33) showed no statistically significant difference between. (2) However, compared with the control group aged 8–9 (N = 39), the ADHD group of the same age (N = 53) showed significantly lower CBF in the left postcentral gyrus and left middle frontal gyrus regions. (3) Further, the ADHD group aged 10–12 (N = 34) demonstrated significantly lower CBF in the left superior occipital region than the age-matched control group (N = 37). These age-specific differences suggest variations in ADHD-related domains during brain development post age 6–7.

## Introduction

Attention deficit hyperactivity disorder (ADHD) is a neurodevelopmental disorder with a prevalence of 5–7.5% among children^1^, and it can persistently affect quality of life from school age to late adolescence. However, the exact cause of ADHD remains not fully understood, despite numerous efforts of previous studies^2^. Most likely preposition of understanding mechanism of ADHD includes genetic and environmental factors which then lead to neurobiological manifestation^3^. Thus, understanding associated changes in brain development may be critically important in treatment and mitigation of ADHD, especially when biomarkers from various technology could elucidate how ADHD symptoms are related to brain structural and functional abnormalities^4^. One of major research areas to further understand neurobiological features is neuroimaging such as structural or functional brain imaging.

In addition to consistent findings of volumetric differences of dorsolateral prefrontal cortex^5^, caudate, pallidum and etc. between ADHD and typically developing children, studies of structural findings of ADHD have demonstrated that individuals with ADHD exhibit delayed development in prefrontal cortex^6–8^, reaching peak cortical thickness at 10.5 years while typically developing children at 7.5 years. Such developmental aspects are also being identified in brain functional research.

Previously, numerous studies examining brain function in ADHD have investigated brain metabolism and subsequent cerebral blood flow (CBF) change. For example, the brain activity in the fronto-parietal cortices of children with ADHD has been shown to differ from that in typically developing children, specifically in the form of hypoactivations in boys and hyperactivations in girls^9^. Meanwhile, resting state fMRI studies have revealed decreased regional homogeneity, which increases with higher brain metabolism, in the fronto-striatal-cerebellar circuits of people with ADHD^10^, and a study using fMRI to examine CBF has demonstrated hypoactivation in the frontal regions and fronto-striatal networks, along with hyperactivation in the posterior regions during cognitive tasks, which is possibly due to a compensatory mechanism, in children and adolescents with ADHD^11^. Therefore, differences in the activity levels of brain regions such as the frontal or parietal cortex have also been demonstrated between children with ADHD and those without.

However, above mentioned previous studies have expounded on general brain function differences of ADHD children of various ages as a whole group, rather than on developmental trajectory, and there is a need for more studies to further investigate dynamic changes of brain function according to age. It can be performed using novel technologies, particularly since brain imaging research is challenging with younger children.

Arterial spin labeled (ASL) perfusion magnetic resonance imaging (MRI) is a noninvasive method that allows for the quantification of blood flow, an important physiological parameter^12^. This method involves continuously inverting proton spins of water coming from arterial blood at the neck area and labeling by observing the effect of this inversion on the strength of brain MRI. The increased use of 3 Tesla clinical MRI systems, along with advances in ASL technology, has dramatically improved the quality of ASL images. However, the quantitative measurement of regional cerebral blood flow (rCBF) with ASL depends on several parameters, including T1 in brain tissue, T1 in arterial blood, and arterial transit time (ATT), which represents the period required for labeled blood to travel from the labeled area to the imaging tissue. It is crucial to consider transit time when measuring absolute rCBF using ASL; thus, ASL methods using multiple post-label delay acquisitions have been developed^13^. Because of its noninvasiveness, ASL is commonly used to improve the precision of 3 Tesla MRI in patients with brain infarction and epilepsy^14,15^.

As a relatively new method of brain imaging in psychiatry, ASL is utilized to quantify brain tissue perfusion by using labeled arterial blood as an endogenous tracer^16^. The ASL method has been reported to induce less across-subject variability and long term reproducibility^17,18^. Moreover, when compared to functional magnetic resonance imaging (fMRI), ASL has been shown to be more sensitive to the tonic changes—rather than the phasic responses—of brain metabolism to a given cognitive task^17,19^.

With this background, ASL has the potential to be a good diagnostic and evaluation test for ADHD, especially when research with younger children is necessitated to observe brain functional changes along with aging. We therefore hypothesized that differences in the CBF of ASL would allow us to observe developmental changes in brain regions associated with ADHD.

## Methods

### Participants

Children with ADHD and a control group of children without ADHD were recruited from the outpatient clinic of the Child and Adolescence Psychiatry Department at Seoul National University as well as from Jung-gu Mental Health Welfare Center in Seoul. The inclusion criteria for the ADHD group were as follows: (1) ages 6–12, (2) previously diagnosed with ADHD according to Diagnostic and Statistical Manual of Mental Disorders fifth edition (DSM-5) and Schedule for Affective Disorders and Schizophrenia for School-Age Children-Present and Lifetime Version (K-SADS-PL). Meanwhile, the inclusion criteria for the control group were as follows: (1) ages 6–12, (2) not diagnosable with any child psychiatric disorder according to psychiatric interviews and K-SADS-PL.

The exclusion criteria for both groups were as follows: (1) Diagnosed with congenital genetic disease, (2) a history of prominent acquired brain injury such as cerebral palsy, (3) diagnosed with epilepsy, neurologic disease, or untreated sensory disorder, (4) history of schizophrenia or psychosis, (5) diagnosed with obsessive–compulsive disorder, major depressive disorder, or bipolar disorder, and (6) diagnosed with language disorder or severe learning disorder. Both groups were also divided into the same three age groups: 6–7, 8–9, and 10–12. Group division into every year would be ideal, but the number of participants was limited in this study. Therefore, 2–3 year grouping of participants was considered reasonable in this study.

The Advanced Test of Attention (ATA), a computerized cognitive test that measures continuous and selective attention and impulsiveness in children and adolescents^20^, was performed on children in both groups for reference. ATA shows age-adjusted T-scores (mean = 50, Standard deviation (SD) = 10) of four indices: omission errors, commission errors, response time (RT), and SD of RTs overall (through the whole task) for each visual and auditory section. ATA is a good diagnostic tool because of its relevance to the characteristics of ADHD, such as inattention and impulsivity. ATA was performed in this study for reference, but the diagnostic division of ADHD and control group was decided with DSM-5 and K-SADS-PL as in general clinical setting.

We recruited 157 children with ADHD (mean age, 8.15 ± 1.75 years; male: 123; female: 34) and 109 children without ADHD (mean age, 8.65 ± 1.68 years; male: 56; female: 53) (Table 1). All of the 157 children in the ADHD group and the 109 children in the control group were classified into subgroups of ages 6–7, ages 8–9, and ages 10–12, and CBF was compared and analyzed between age groups. In the ADHD group, there were 70 participants aged 6–7, 53 aged 8–9, and 34 aged 10–12; in the control group, there were 33 participants aged 6–7, 39 aged 8–9, and 37 aged 10–12 (Table 2). There were 10 ADHD participants with tic disorder, 5 ADHD participants with oppositional defiant disorder (ODD), and 9 ADHD participants and 1 control participant with other mild mood or anxiety disorder (Table 1). Also, there was 1 ADHD participant who was taking medication during the study. The portion of participants with tic disorder or ODD as comorbidities and of participants taking medication was considered negligible during analysis.

**Table 1:** Characteristics of ADHD group and control group.

	ADHD group	Control group	*p*
Total	157	109	
Gender, *n* (%)			< .001
Male	123 (78.3)	56 (51.4)	
Female	34 (21.7)	53 (48.6)	
Age, mean (SD)	8.15 (1.75)	8.65 (1.68)	.009
FSIQ			
Number of missing value, n (%)	18 (11.5)	6 (5.5)	
Min	70	69	
Max	136	142	
Mean (SD)	104.02 (14.76)	111.48 (14.76)	.391
ATA, mean (SD)			
Visual omission	68.85 (20.64)	58.54 (18.54)	< .001
Visual commission	65.58 (20.21)	57.24 (17.51)	< .001
Auditory omission	66.72 (19.33)	59.69 (18.73)	< .001
Auditory commission	61.88 (18.03)	56.52 (16.72)	.009

*ADHD* attention deficit hyperactivity disorder, *ATA* Advanced test of attention, *SD* standard deviation, *FSIQ* full scale intellectual quotient.

**Table 2:** Characteristics of ADHD group and control group according to age group.

	6–7		*p*-value	8–9		*p*-value	10–12			*p*-value	
	ADHD	Control		ADHD	Control		ADHD		Control		
Total	70	33		53	39		34		37		
Gender, *n* (%)			.001			.062				.004	
Male	54 (77.1)	15 (45.5)		42 (79.2)	24 (61.5)		27 (79.4)		17 (45.9)		
Female	16 (22.9)	18 (54.5)		11 (20.8)	15 (38.5)		7 (20.6)		20 (54.1)		
Age, mean (SD)	6.61 (.49)	6.64 (.49)	.830	8.42 (.50)	8.56 (.50)	.160	10.88 (.88)		10.54 (.77)	.090	
FSIQ (number of missing values)	(5)			(5)	(2)		(8)		(4)		
Min	70	69		70	82		76		88		
Max	132	142		136	142		131		132		
Mean (SD)	104.74 (14.82)	112.52 (17.96)	.155	103.00 (16.67)	111.35 (14.22)	.209	104.12 (15.89)		110.58 (11.93)	.054	
ATA, mean (SD)											
Visual omission	73.93 (19.30)	66.15(22.58)	.043	70.15 (22.24)	58.41 (17.02)	.012	56.38 (15.43)		51.89 (13.16)	.044	
Visual commission	70.27 (20.56)	58.91 (17.00)	.008	64.92 (20.40)	60.28 (19.50)	.131	56.94 (16.36)		52.54 (15.06)	.127	
Auditory omission	65.03 (17.64)	62.09 (20.60)	.323	66.88 (20.27)	57.10 (16.37)	.005	69.44 (20.93)		60.35 (19.53)	.046	
Auditory commission	58.48 (16.93)	53.66 (15.04)	.191	66.53 (19.53)	58.90 (18.47)	.047	60.88 (16.52)		56.49 (16.20)	.066	

*ADHD* attention deficit hyperactivity disorder, *ATA* Advanced test of attention, *SD* standard deviation, *FSIQ* full scale intellectual quotient.

Informed consent was obtained both from every participant and from their parent and/or legal guardian after study design was explained. This study was approved by the Institutional Review Board of Seoul National University Hospital (IRB approval number: 2008-116-1150, 1507-118-690, 1206-054-414). This study was performed in accordance with the Declaration of Helsinki.

### MRI imaging protocol

All participants were scanned at Seoul National University Hospital using a 3 T Tim Trio MRI machine (Siemens Healthcare, Erlangen, Germany) with a 32-channel head coil. Pulsed arterial spin labeling (PASL) data were acquired with the PICORE Q2TIPS sequence^21^ using the following protocol: 80 pairs of label/control ASL images were acquired in a transversal orientation at a single inversion time of 1800 ms (EPI-readout, TR = 3000 ms, TE = 12 ms, TI1 = 700 ms, label thickness = 100 mm, PICORE Q2T perfusion mode, voxel resolution: 3 × 3 × 5 mm, flip angle = 90°, time of acquisition = 4:06 min, echo planar imaging (EPI)-factor = 49, partial fourier = 6/8, Bandwidth = 3004 Hz/Pixel). A calibration image with identical readout parameters with no background suppression or ASL labeling was automatically collected within the same scan. A 3D T1-weighted structural image (magnetization-prepared rapid acquisition gradient echo (MPRAGE): voxel resolution 1.0 × 1.0 × 1.0 mm, FoV = 256 mm, TR = 2100 ms, TE = 3.71 ms, flip angle = 10°, time of acquisition = 4:52 min) was also acquired (Table S1).

### Preprocessing

A preprocessing procedure was performed for all data using the Oxford Centre for Functional MRI of the Brain (FMRIB)’s Software Library (FSL)^22^. Tissue segmentation of the structural T1-weighted image was processed using the fsl_anat pipeline. The fsl_anat pipeline performed brain extraction with the brain extraction tool (BET)^23^ while performing gray and white matter tissue segmentation using a partial volume estimate (PVE)^24^. Finally, the structural image was registered to the Montreal Neurological Institute (MNI) standard space with a linear and non-linear registration step of the fsl_anat pipeline.

ASL data were processed using the oxford_asl command line utility of the Bayesian Inference for Arterial Spin Labeling (BASIL) toolbox^25^ within FSL. In the first step, the ASL image was registered to the processed T1 structural image with the M0 image of ASL, including motion correction. In the second step, a deformation field that was generated from the registration step between ASL and T1 structural image was applied to MNI space registration. Finally, a quantitative CBF image was calculated using normalized ASL data on MNI standard space for group analysis.

### Group analysis

The corrected *p*-value for control family-wise error rate (FWEp < 0.05) was assigned to the group comparison between the ADHD and control groups with 5000 permutations using the Permutation Analysis of Linear Models (PALM) toolbox^26^.

A group comparison by age between the ADHD and control groups was performed with the Statistical Parametric Mapping 12 Toolbox (SPM12) (http://www.fil.ion.ucl.ac.uk)^27^. The data of each group were further separated into three subgroups by age range (6–7 years, 8–9 years, and 10–12 years) for age-matched analyses. Random-effect analysis was applied for multiple comparisons by age with a full factorial design. Statistical results were corrected by family-wise error correction (FWEp < 0.05). Age, sex, and intellectual quotient (IQ) were adjusted for as covariates in between-group comparison. In different age groups, sex and IQ were considered as covariates. Activated region labeling of each group comparison was applied with automated anatomical labeling atlas 3 (AAL v3)^28^.

## Result

### Participant characteristics.

The characteristics of the participants in the ADHD group and the control group are presented below (Tables 1 and 2).

### ADHD versus control in the whole group

**Figure 1:**
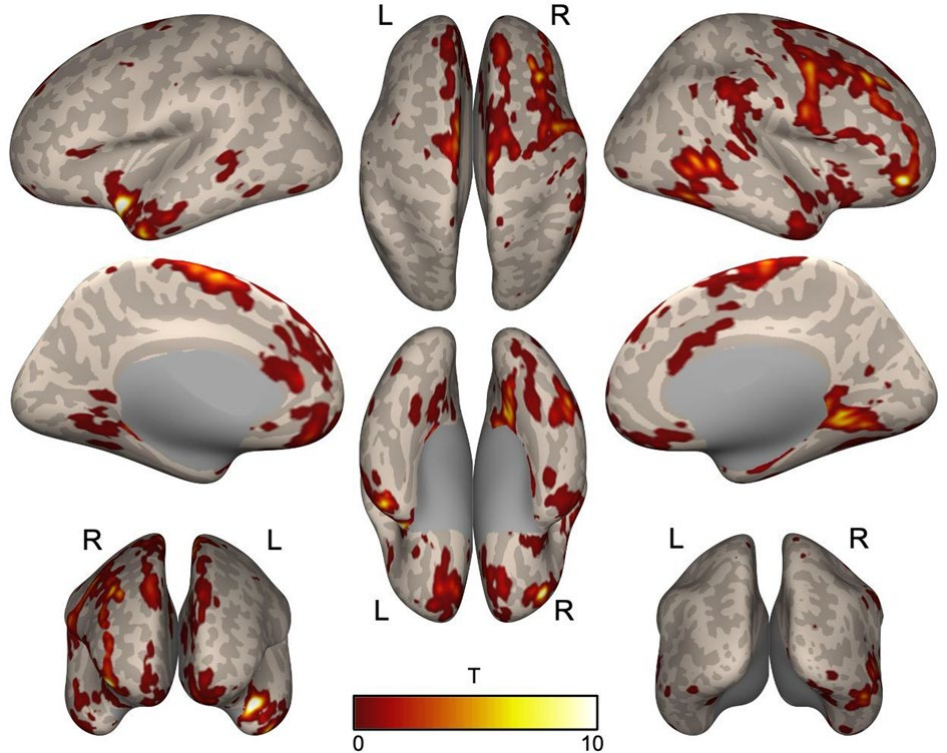
Between-group comparison (HC vs. ADHD). The ADHD group exhibited lower activity in the left superior temporal gyrus and right middle frontal gyrus regions than the control group.

Analysis of the CBF through ASL MRI of the 157 participants with ADHD and the 109 control participants showed that, overall, the ADHD group exhibited lower CBF in the left superior temporal gyrus and right middle frontal gyrus regions than the control group (Table 3, Fig. 1).

**Table 3:** Between group comparison (HC vs. ADHD).

HC versus ADHD	t-value	MNI Coordinates		
Region label		x	y	z
OFCant_R	19.854	44	48	− 18
Temporal_Pole_Sup_L	19.251	− 40	0	− 18
Frontal_Mid_2_R	14.102	42	34	44
Precentral_R	9.831	54	6	50
Frontal_Mid_2_R	8.384	46	56	6
Supp_Motor_Area_L	7.450	− 4	2	70
Temporal_Inf_R	7.317	48	− 54	− 10
Temporal_Mid_R	7.071	62	− 42	2
Lingual_R	7.046	16	− 48	− 2
OFCmed_L	5.622	− 14	46	− 24
SupraMarginal_R	5.459	64	− 34	48
Frontal_Sup_Medial_R	5.090	11	72	9
Cingulate_Ant_L	4.698	− 6	42	10
Rectus_L	4.134	− 4	46	− 26
Caudate_L	3.870	− 18	22	8
Temporal_Pole_Sup_R	3.850	64	4	− 4
Putamen_R	3.341	26	18	2
Parietal_Sup_R	3.112	18	− 62	72
OFCant_L	3.112	18	− 62	72
Parietal_Sup_R	3.312	56	− 34	58
Temporal_Pole_Mid_R	3.000	44	18	− 42
Cerebellum_3_L	9.289	− 10	− 38	− 20
Cerebelum_8_R	23.599	40	− 62	− 58
Cerebellum_8_L	5.412	− 26	− 42	− 54
Cerebelum_Crus2_L	34.286	− 42	− 68	− 52

*ADHD* attention deficit hyperactivity disorder, *HC* Healthy control.

### ADHD versus control according to age group

#### ADHD aged 6–7 versus control of all age groups

**Figure 2:**
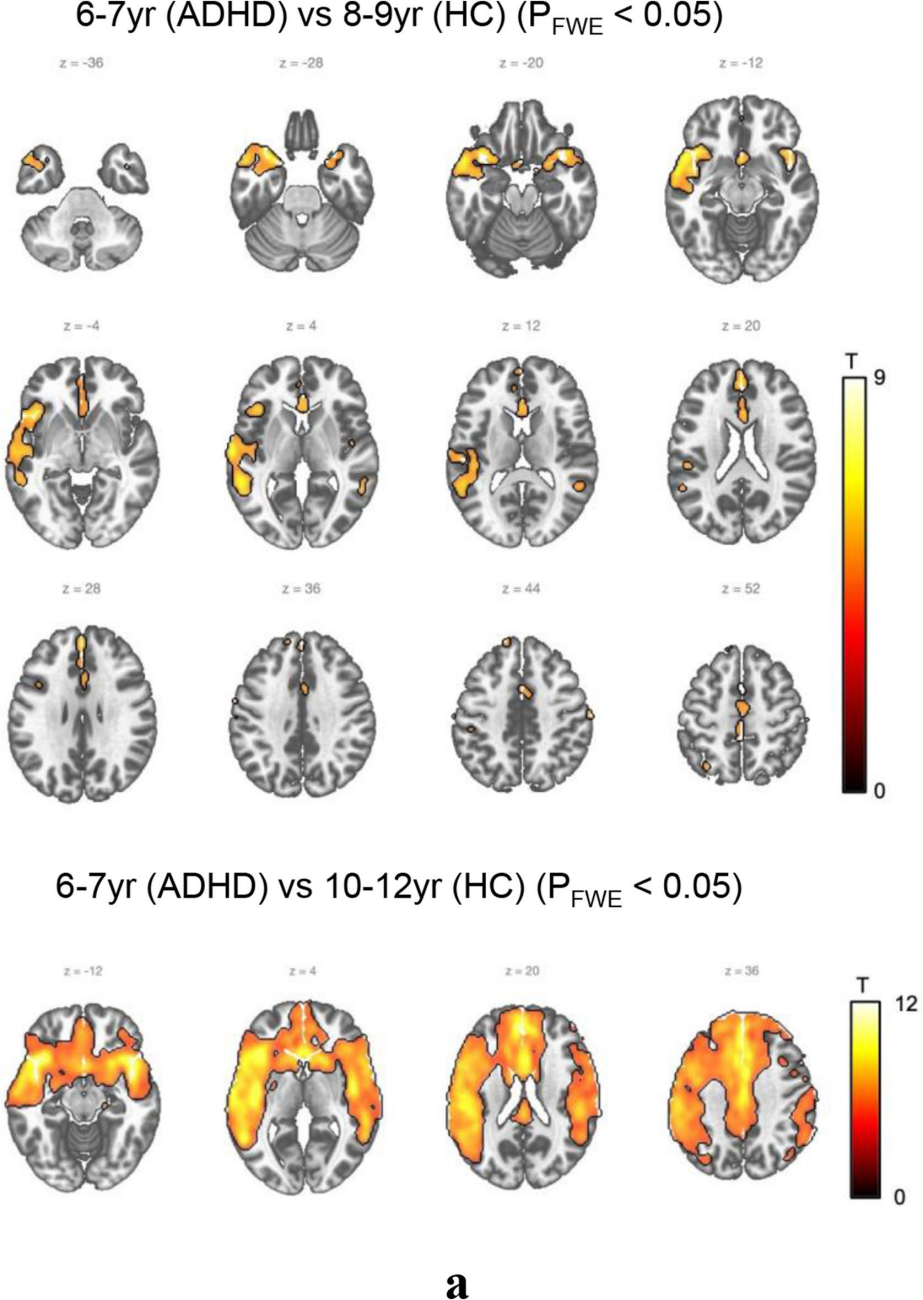

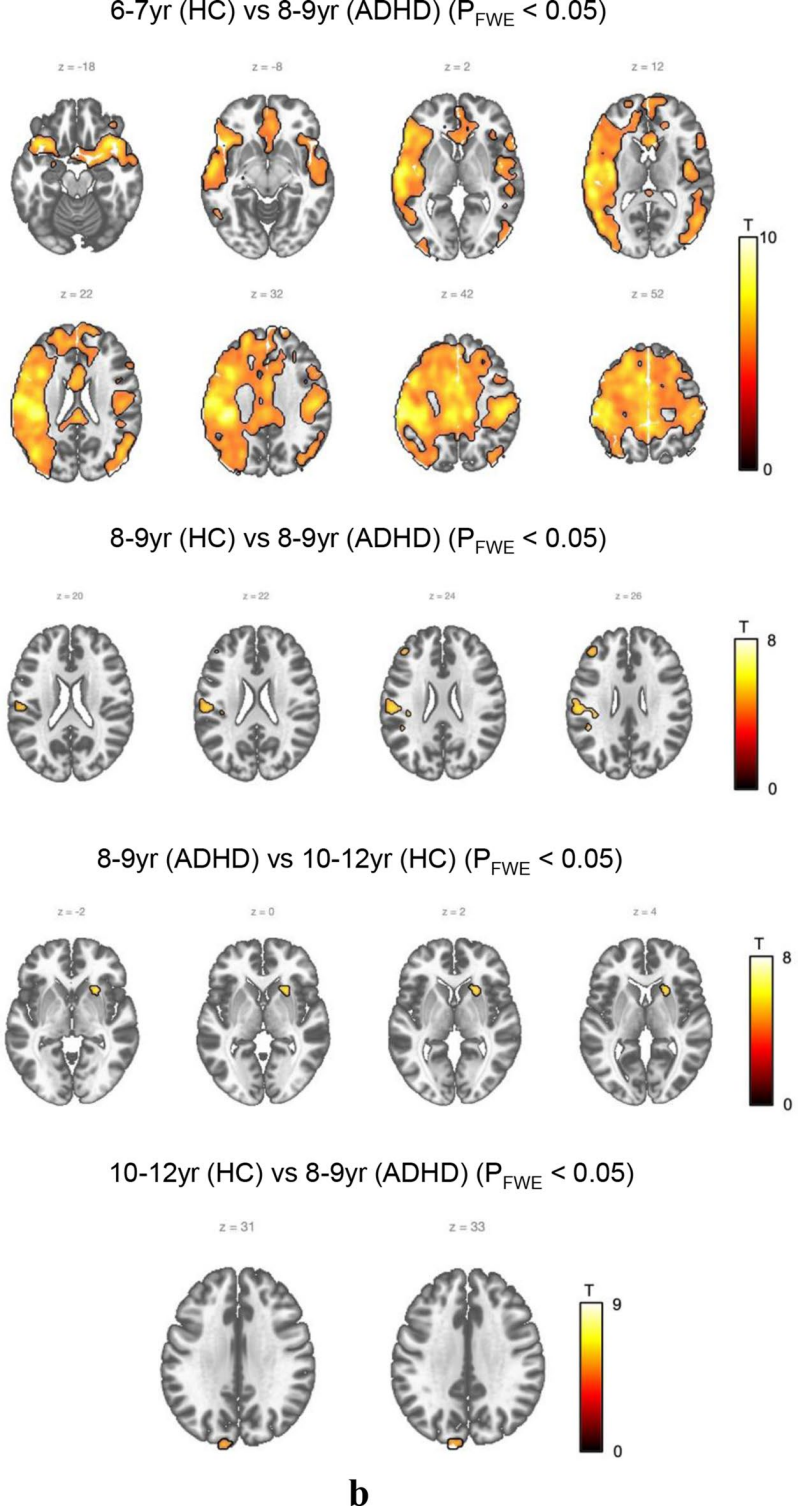

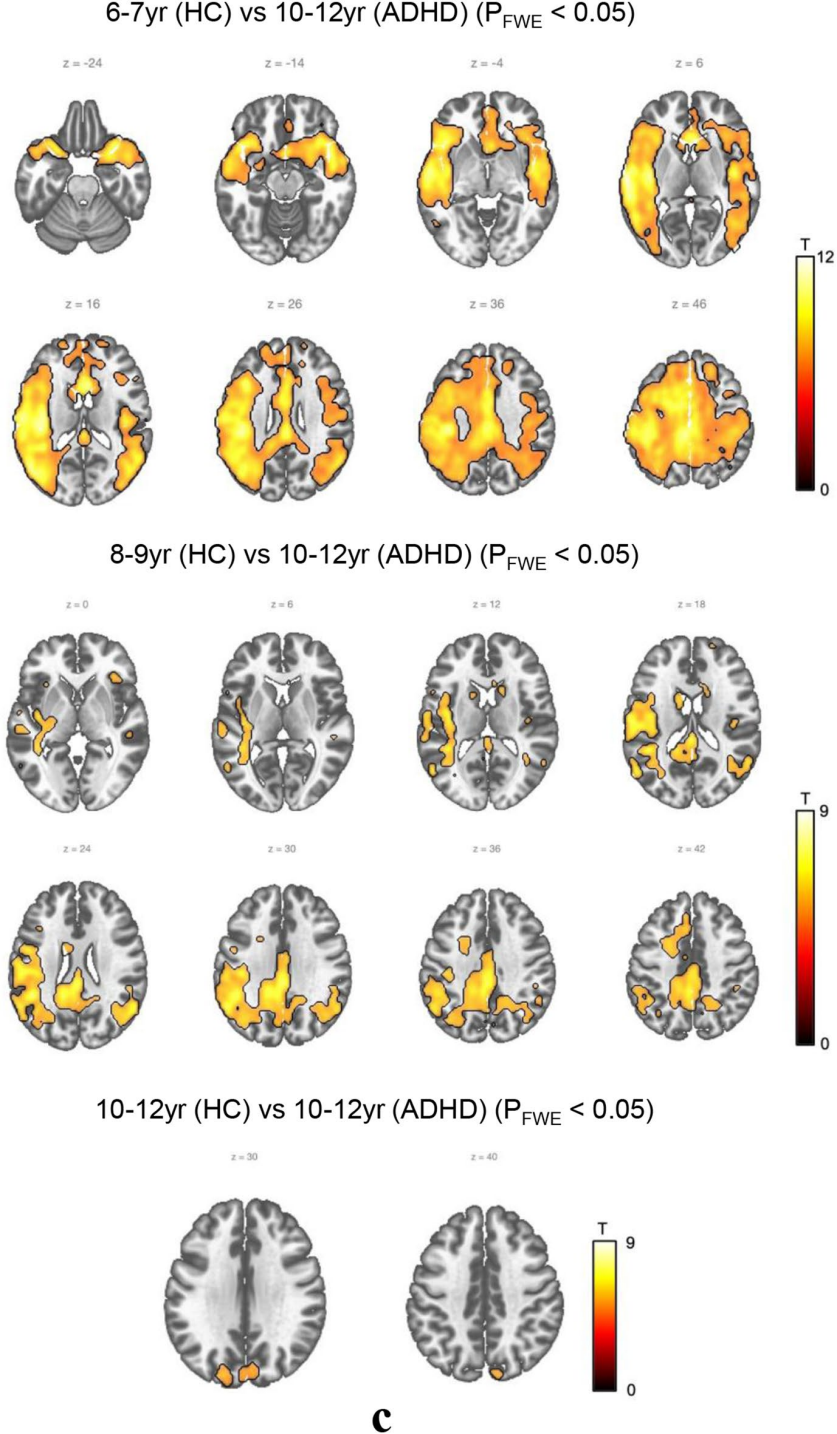
Between-group comparison by age (HC vs. ADHD). (**a**) ADHD (6-7 yr) versus Control. No statistically significant difference was observed between the ADHD group and the control group at the same age of 6–7. However, between the ADHD group aged 6–7 and the control group aged 8–9, the ADHD group aged 6–7 demonstrated higher activity in the left superior temporal gyrus and left middle temporal gyrus regions. Meanwhile, between the ADHD group aged 6–7 and the control group aged 10–12 years, the ADHD group aged 6–7 showed higher activity in the right insula, left superior frontal gyrus, medial, and left inferior frontal gyrus pars orbitalis. (**b**) ADHD (8–9 yr) versus Control. Between the ADHD group aged 8–9 and the control group aged 6–7, the ADHD group aged 8–9 showed lower activity in the left postcentral gyrus and left supramarginal gyrus, along with greater such activity than the control group aged 6–7. Moreover, when comparing the ADHD group aged 8–9 and the control group aged 8–9, the age-matched ADHD group showed less activity in the left postcentral gyrus and left middle frontal gyrus regions. In comparing the ADHD group aged 8–9 with the control group aged 10–12, the activity of the ADHD group aged 8–9 was found to be increased in the right putamen region whereas it was decreased in the left superior occipital region. (**c**) ADHD (10–12 yr) versus Control. When comparing the ADHD group aged 10–12 and the control group aged 6–7, the ADHD group aged 10–12 showed less activity in the areas of the left superior temporal gyrus, right precuneus, and left middle temporal gyrus. When compared with the control group aged 8–9, the ADHD group aged 10–12 showed less activity in the left supramarginal and left posterior cingulate regions, while the ADHD group aged 10–12 also showed less activity in the left superior occipital region compared to the control group aged 10–12.

We began by conducting ASL MRI comparison between the ADHD group aged 6–7 and the control group of all ages (Fig. 2a, S1.a, S1.b).

No statistically significant difference of CBF was observed between the ADHD group and the control group at the same age of 6–7. However, between the ADHD group aged 6–7 and the control group aged 8–9, the ADHD group aged 6–7 demonstrated higher CBF in the left superior temporal gyrus and left middle temporal gyrus regions. Meanwhile, between the ADHD group aged 6–7 and the control group aged 10–12 years, the ADHD group aged 6–7 showed higher CBF in the right insula, left superior frontal gyrus, medial, and left inferior frontal gyrus pars orbitalis.

#### ADHD aged 8–9 versus control of all age groups

Next, we conducted a comparison between the ADHD group aged 8–9 and the control group of all ages (Fig. 2b, S1.c, S1.d, S1.e).

Between the ADHD group aged 8–9 and the control group aged 6–7, the ADHD group aged 8–9 showed lower CBF in the left postcentral gyrus and left supramarginal gyrus. Moreover, when comparing the ADHD group aged 8–9 and the control group aged 8–9, the age-matched ADHD group showed lower CBF in the left postcentral gyrus and left middle frontal gyrus regions. In comparing the ADHD group aged 8–9 with the control group aged 10–12, the CBF of the ADHD group aged 8–9 was found to be increased in the right putamen region whereas it was decreased in the left superior occipital region.

#### ADHD aged 10–12 versus control of all age groups

Last, we conducted a comparison between the ADHD group aged 10–12 and the control group of all ages (Fig. 2c, S1.f, S1.g, S1.h).

These results showed that, when comparing the ADHD group aged 10–12 and the control group aged 6–7, the ADHD group aged 10–12 showed lower CBF in the areas of the left superior temporal gyrus, right precuneus, and left middle temporal gyrus.

Moreover, when compared with the control group aged 8–9, the ADHD group aged 10–12 showed lower CBF in the left supramarginal and left posterior cingulate regions, while the ADHD group aged 10–12 also showed lower CBF in the left superior occipital region compared to the control group aged 10–12.

## Discussion

This study not only reapproved functional alteration of ADHD^29^ compared to control group, as shown in previous studies such as that of Luo et al. using default mode network (DMN) analysis, but also identified certain differences in the activity of the left superior temporal gyrus and right middle frontal gyrus between ADHD and control groups, as shown in other previous research^30^. The difference in right middle frontal gyrus was one of the main findings of this study, and in the same context, other resting state fMRI studies, especially using DMN, have focused on altered function of the frontal cortex which was known to be related with the executive function of ADHD^31^. Our results also found statistically significant differences in CBF across age groups between the ADHD and control groups. Regional differences of CBF revealed in between age group analysis were disposed across whole area of brain: for example, in left postcentral gyrus, left supramarginal, left superior occipital region, etc. These regions demonstrating significant functional differences included postcentral gyrus, which, in charge of sensorimotor function, has been known to be related to ADHD, especially with decreased cortical thickness^32^. Other distribution could be explained with a common hypothesis from previous studies that focus of ADHD pathology should be shifted from regional abnormalities to distributed network^31,33^, so future studies could aim to discover detailed networks within regions and their relations with ADHD.

Another key finding was a significant change in the brain activation trajectory between ages 6–7 and 8–12. Specifically, the ADHD group aged 6–7 did not show any significant differences in CBF compared to the control group of the same age, rather exhibited increased CBF compared to the control group aged 8–12 in specific brain regions such as the superior and middle temporal gyri, even though the overall activity of the ADHD group was lower than that of the control group.

These findings align with prior research highlighting the occurrence of delayed maturation in ADHD. For instance, Tang et al. demonstrated delayed maturation of brain networks in an ADHD group using resting fMRI^34^. In another study, Yasumura et al.^35^ concluded that ADHD children, compared to typically developing children, exhibited less age-related changes of the right and middle prefrontal cortex (PFC), with the left PFC compensating. Another study using diffusion tensor imaging (DTI) comparing drug-naive children with ADHD with adults with ADHD found that only the adults with ADHD demonstrated white matter alteration, suggesting a later childhood developmental delay of white matter^36^. However, due to the absence of sophisticated age division in childhood, the exact period of delay has yet to be specified, and there have been few studies that have attempted to discover the age at which this turning point in ADHD brain development occurs. One of scarce number of research focusing on specific time point was a study using single photon emission computed tomography (SPECT) which found the existence of increasing prefrontal regional cerebral blood flow lateralization with age in ADHD along with a different developmental trajectory for prefrontal asymmetry in children with ADHD aged 7 or older^37^, which corresponds to the results of our study.

Our study, considered in the context of the above-mentioned research, reaffirmed the unique brain developmental pattern that occurs in ADHD by using ASL as well as considering a more delicate age group division than previous studies. ASL may thus prove useful for inferring the timing of brain development in brain areas associated with ADHD, particularly in younger children. This is because, in contrast to SPECT, ASL involves no radiation exposure, making it non-invasive, and it also has a short test time of approximately 5 min, which is also particularly advantageous for children. ASL also provides several advantages over blood oxygenation level-dependent MRI (BOLD MRI), which is another method that is extensively used to investigate CBF. For example, ASL could yield better spatial localization and signal quantification. Further, due to its frequency-independent power spectrum, ASL is more suitable for tracking slow varying changes in the brain^38^. To summarize, ASL is advantageous due to its non-invasiveness, short test time, and sensitivity to tonic changes in brain metabolism, thus offering potential as a diagnostic and evaluative tool for ADHD.

Exploiting these benefits of ASL, this study could serve as a starting point for further elucidating brain developmental differences between ADHD and more typically developing children. As mentioned above, there have been numerous studies focused on the biological features of ADHD that have used various methods, and the present study attempted to suggest findings that could be obtained using relatively new techniques and that could be useful for children. We also presented ages 6–7 as a possible period wherein there is an accelerating brain activity disparity between ADHD and typically developing children.

However, this study still has several limitations. First, the sample size of our study was relatively small. Further studies with larger sample sizes are thus needed to better establish the diagnostic value of ASL. Secondly, the inclusion of more covariates, such as neuropsychological tests and subtypes of ADHD, could be applied in the analysis to reflect CBF changes more accurately according to individual ADHD characteristics. We did not include medication status and comorbidities including tic disorder and oppositional defiant disorder, which are commonly associated with ADHD, as covariates to statistically control during analysis, since the portion of participants taking medication, or with comorbidities were negligible. However, in future studies with a large number of various participants, these variables could be more sophisticatedly controlled.

Third, as in other research with children and adolescents with ADHD, our study results were male-dominated. Despite inevitability of these results due to larger prevalence of ADHD in male^5^, sex differences in developmental change of ADHD should be further contemplated. During childhood and adolescence, males are known to show more decline of cerebral blood flow^39^, and a few studies considered sex differences in research on regional cerebral blood flow^9^. Our study did adjust sex differences for ASL analysis, but recruiting more female samples could later render a more precise study of developmental trajectory of ADHD according to sex.

In addition, it is necessary to further investigate the correlation between the area with the difference in ASL activation and the area related to ADHD in future studies. For example, striatum could be suggested as a focus of imaging study as reward processing has been proposed to contribute to ADHD^40^.

Also, it would be helpful to conduct symptom-related analysis with such imaging study, for example, ADHD scale related regression analysis. In this context, ASL may also be useful in evaluating changes of symptoms or therapeutic effects, which would allow us to gauge the appropriate timing of treatment and evaluation, since we know from the current study that ADHD is associated with development changes in certain periods.

In summary, future studies involving larger sample sizes and more covariates are necessary to delineate the developmental trajectory of ADHD more precisely and to enhance the diagnostic capabilities of ASL MRI in the understanding and management of ADHD.

## Conclusion

This study observed a difference in CBF of ADHD-related domains between an ADHD group and a control group. Moreover, when comparing within and between groups by age, it was found that the difference in the CBF varied. Notably, the most substantial difference in activity was observed between the ages of 6–7 and 8–12. This suggests not only that the development of ADHD-related domains in the brain development process occurs most significantly between 6–7 and 8–9 years of age but also that ASL could be employed in developmental studies of ADHD children.

These findings could be utilized in enhanced understanding of developmental trajectory of ADHD with future research with additional covariates and imaging-symptom relation study.

## Supplementary Information

Supplementary Table S1.

## Data Availability

The datasets used and/or analyzed during the current study available from the corresponding author on reasonable request.
