# Author Correction: Comparison of arterial spin labeled MRI (ASL MRI) between ADHD and control group (ages of 6–12)

**DOI:** 10.1038/s41598-025-92290-4

**Published:** 2025-04-03

**Authors:** You Bin Lim, Huijin Song, Hyunjoo Lee, Seungbee Lim, Seo Young Kwon, Jeeyoung Chun, Sujin Kim, Ceren Tosun, Kyung Seu Yoon, Chul-Ho Sohn, Bung-Nyun Kim

**Affiliations:** 1 Division of Child and Adolescent Psychiatry, Department of Psychiatry, Seoul National University College of Medicine, 101 Daehak-ro, Jongno-gu, Seoul, 03080 Republic of Korea; 2 Biomedical Research Institute, Seoul National University Hospital, Seoul, Republic of Korea; 3 Istanbul University-Cerrahpasa Medical Faculty Child and Adolescent Psychiatry, Istanbul, Turkey; 4 Department of Radiology, Seoul National University College of Medicine, 101 Daehak-ro, Jongno-gu, Seoul, 03080 Republic of Korea; 5 Department of Psychiatry, Hanyang University Hospital, Seoul, Republic of Korea

Correction to: *Scientific Reports* 10.1038/s41598-024-63658-9, published online 28 June 2024

The Acknowledgments section in the original version of this Article was incomplete.

“This research was supported by the National Research Foundation (NRF) funded by the Korean Government (MSIT) (2020M3E5D9080787 to B-NK), and by a grant of the R&D project, funded by the National Center for Mental health (MHER22A01).”

now reads:

“This research was supported by the National Research Foundation (NRF), funded by the Korean Government (MSIT) (2020M3E5D9080787 to B-NK, No. RS-2024-00397737), and by a grant from the R&D project, funded by the National Center for Mental Health (MHER22A01).”

The original Article has been corrected.

