# Supplementary material for: Comparison of arterial spin labeled MRI (ASL MRI) between ADHD and control group (ages of 6–12)
Source: Sci Rep. 2024 Jun 28;14:14950. doi: 10.1038/s41598-024-63658-9 (PMC11213899; doi:10.1038/s41598-024-63658-9)
Supplement: Supplementary file 1 — Supplementary Table S1. [file 41598_2024_63658_MOESM1_ESM.docx]

**Supplementary**

**Supplementary S1. Between group comparison by age (HC vs ADHD)**

**Supplementary S1.a 6-7yr (ADHD) vs 8-9yr (HC)**

| 6-7yr (ADHD) vs 8-9yr (HC) |  | MNI Coordinates | | |
| --- | --- | --- | --- | --- |
| Region Label | t-value | x | y | z |
| Temporal_Mid_L | 7.046 | -56 | 4 | -16 |
| Temporal_Sup_L | 6.942 | -64 | -14 | 4 |
| Frontal_Inf_Orb_2_L | 6.919 | -42 | 18 | -10 |
| OFCpost_L | 6.836 | -26 | 12 | -26 |
| Rolandic_Oper_L | 5.726 | -44 | -30 | 14 |
| Insula_R | 6.591 | 40 | 16 | -16 |
| Frontal_Sup_Medial_L | 6.341 | 0 | 50 | 28 |
| Supp_Motor_Area_L | 6.157 | -14 | -8 | 62 |
| Cingulate_Mid_R | 5.637 | 4 | 6 | 40 |
| Paracentral_Lobule_L | 5.557 | -2 | -30 | 56 |
| Supp_Motor_Area_L | 5.335 | -2 | 8 | 68 |
| Cingulate_Ant_L | 5.221 | -2 | 48 | -2 |
| Precuneus_L | 5.805 | -8 | -50 | 72 |
| Parietal_Sup_L | 5.712 | -34 | -62 | 54 |
| Precuneus_R | 5.712 | 10 | -44 | 74 |
| Postcentral_R | 5.592 | 64 | -14 | 42 |
| Temporal_Mid_R | 5.511 | 56 | -50 | 6 |
| Parietal_Sup_R | 5.388 | 18 | -62 | 72 |
| Frontal_Inf_Oper_L | 5.192 | -40 | 12 | 30 |
| Postcentral_L | 5.184 | -60 | -4 | 40 |
| Parietal_Inf_R | 5.094 | 58 | -32 | 56 |
| Precuneus_R | 5.071 | 4 | -46 | 62 |
| Parietal_Inf_L | 5.027 | -46 | -28 | 44 |
| Postcentral_L | 4.989 | -60 | -18 | 38 |
| Temporal_Pole_Mid_R | 4.983 | 40 | 4 | -34 |

*ADHD* attention deficit hyperactivity disorder, *HC* Healthy control.

**Supplementary S1.b 6-7yr (ADHD) vs 10-12yr (HC)**

| 6-7yr (ADHD) vs 10-12yr (HC) |  | MNI Coordinates | | |
| --- | --- | --- | --- | --- |
| Region Label | t-value | x | y | z |
| Frontal_Sup_Medial_L | 10.885 | -4 | 48 | 22 |
| Insula_R | 10.790 | 40 | 18 | -16 |
| Frontal_Inf_Orb_2_L | 10.536 | -40 | 20 | -10 |
| Rolandic_Oper_L | 10.217 | -44 | -20 | 12 |
| Frontal_Sup_Medial_L | 10.159 | -2 | 32 | 44 |
| Supp_Motor_Area_L | 9.957 | -4 | 10 | 44 |
| Temporal_Mid_L | 9.770 | -54 | -52 | 4 |
| Paracentral_Lobule_L | 9.717 | -2 | -26 | 56 |
| Temporal_Sup_L | 9.643 | -64 | -12 | 6 |
| Frontal_Mid_2_L | 9.605 | -24 | 10 | 54 |
| Insula_R | 9.313 | 46 | -6 | 2 |
| Parietal_Inf_L | 8.711 | -44 | -50 | 52 |
| Postcentral_L | 8.677 | -60 | -16 | 28 |
| Temporal_Sup_L | 8.670 | -54 | 4 | -14 |
| Caudate_R | 8.601 | 18 | 18 | 2 |
| Precuneus_L | 8.599 | -6 | -46 | 72 |
| Temporal_Mid_R | 8.460 | 56 | -36 | 2 |
| Frontal_Inf_Oper_L | 8.422 | -54 | 14 | 20 |
| Precentral_L | 8.194 | -44 | 4 | 50 |
| Paracentral_Lobule_L | 8.161 | -18 | -10 | 64 |
| Parietal_Inf_L | 8.072 | -50 | -32 | 42 |
| Parietal_Inf_R | 8.049 | 56 | -52 | 52 |
| Caudate_L | 7.909 | -10 | 2 | 20 |
| Frontal_Med_Orb_L | 7.883 | -4 | 42 | -8 |
| Postcentral_R | 7.803 | 54 | -26 | 48 |
| Frontal_Sup_2_R | 7.668 | 22 | 32 | 48 |
| Parietal_Sup_R | 5.406 | 14 | -60 | 74 |

*ADHD* attention deficit hyperactivity disorder, *HC* Healthy control.

**Supplementary S1.c 6-7yr (HC) vs 8-9yr (ADHD)**

| 6-7yr (HC) vs 8-9yr (ADHD) |  | MNI Coordinates | | |
| --- | --- | --- | --- | --- |
| Region Label | t-value | x | y | z |
| Postcentral_L | 8.541 | -60 | -14 | 26 |
| Frontal_Sup_2_L | 7.410 | -20 | 52 | 28 |
| Frontal_Mid_2_L | 7.235 | -22 | 26 | 52 |
| Temporal_Sup_L | 8.566 | -62 | -14 | 4 |
| Parietal_Inf_L | 7.161 | -34 | -46 | 46 |
| Temporal_Mid_R | 7.077 | 46 | -72 | 18 |
| Insula_L | 6.913 | -32 | 22 | 6 |
| Frontal_Mid_2_L | 6.889 | -28 | 8 | 60 |
| Frontal_Sup_2_R | 6.764 | 22 | 32 | 46 |
| Insula_R | 6.632 | 42 | -6 | 8 |
| Amygdala_L | 5.457 | -26 | -4 | -16 |
| OFCant_R | 5.445 | 38 | 38 | -18 |
| Precentral_R | 5.116 | 20 | -18 | 72 |
| Parietal_Sup_L | 4.928 | -18 | -50 | 76 |

*ADHD* attention deficit hyperactivity disorder, *HC* Healthy control.

**Supplementary S1.d 8-9yr (HC) vs 8-9yr (ADHD)**

| 8-9yr (HC) vs 8-9yr (ADHD) |  | MNI Coordinates | | |
| --- | --- | --- | --- | --- |
| Region Label | t-value | x | y | z |
| Postcentral_L | 5.752 | -54 | -22 | 26 |
| Frontal_Mid_2_L | 5.179 | -40 | 40 | 28 |
| SupraMarginal_L | 4.883 | -44 | -42 | 26 |
| Cingulate_Mid_L | 4.839 | -2 | -40 | 42 |

*ADHD* attention deficit hyperactivity disorder, *HC* Healthy control.

**Supplementary S1.e 8-9yr (ADHD) vs 10-12yr (HC)**

| 8-9yr (ADHD) vs 10-12yr (HC) |  | MNI Coordinates | | |
| --- | --- | --- | --- | --- |
| Region Label | t-value | x | y | z |
| Putamen_R | 5.747 | 24 | 18 | -2 |
| 10-12yr (HC) vs 8-9yr (ADHD) |  | MNI Coordinates | | |
| Region Label | t-value | x | y | z |
| Occipital_Sup_L | 5.511 | -12 | -92 | 34 |

*ADHD* attention deficit hyperactivity disorder, *HC* Healthy control.

**Supplementary S1.f 6-7yr (HC) vs 10-12yr (ADHD)**

| 6-7yr (HC) vs 10-12yr (ADHD) |  | MNI Coordinates | | |
| --- | --- | --- | --- | --- |
| Region Label | t-value | x | y | z |
| Temporal_Sup_L | 11.629 | -62 | -16 | 4 |
| Precuneus_R | 10.990 | 2 | -42 | 54 |
| Temporal_Mid_L | 10.685 | -42 | -68 | 20 |
| Frontal_Sup_2_L | 10.361 | -22 | -2 | 54 |
| Temporal_Pole_Sup_R | 9.925 | 44 | 8 | -18 |
| Frontal_Inf_Orb_2_L | 9.888 | -42 | 20 | -10 |
| Temporal_Mid_R | 9.637 | 54 | -56 | 20 |
| OFCpost_L | 8.948 | -26 | 12 | -24 |
| Parietal_Inf_L | 8.662 | -34 | -62 | 52 |
| Frontal_Inf_Oper_L | 8.634 | -56 | 8 | 14 |
| Precentral_L | 8.612 | -46 | 6 | 42 |
| Temporal_Sup_R | 8.392 | 56 | -20 | -6 |
| Postcentral_R | 8.232 | 46 | -28 | 44 |
| Frontal_Sup_2_R | 8.178 | 20 | 34 | 44 |
| Occipital_Mid_R | 8.150 | 38 | -80 | 16 |
| Precuneus_L | 7.956 | -12 | -38 | 68 |
| Frontal_Sup_2_R | 6.858 | 18 | 58 | 24 |

*ADHD* attention deficit hyperactivity disorder, *HC* Healthy control.

**Supplementary S1.g 8-9yr (HC) vs 10-12yr (ADHD)**

| 8-9yr (HC) vs 10-12yr (ADHD) |  | MNI Coordinates | | |
| --- | --- | --- | --- | --- |
| Region Label | t-value | x | y | z |
| SupraMarginal_L | 7.631 | -44 | -44 | 24 |
| Cingulate_Post_L | 7.397 | 0 | -32 | 32 |
| Postcentral_L | 7.245 | -56 | -20 | 20 |
| Precuneus_R | 7.233 | 4 | -42 | 54 |
| Insula_L | 7.155 | -38 | -8 | -4 |
| Angular_R | 6.969 | 54 | -58 | 24 |
| Temporal_Mid_L | 6.660 | -54 | -64 | 18 |
| Precentral_L | 6.572 | -48 | 0 | 24 |
| Supp_Motor_Area_L | 6.438 | -4 | 20 | 48 |
| Occipital_Mid_L | 6.265 | -36 | -68 | 32 |
| Caudate_L | 6.242 | -12 | 4 | 20 |
| Parietal_Inf_L | 6.157 | -42 | -50 | 48 |
| Cingulate_Mid_R | 6.057 | 4 | -10 | 32 |
| SupraMarginal_R | 5.871 | 50 | -38 | 28 |
| Precuneus_R | 5.621 | 8 | -70 | 50 |
| Frontal_Inf_Orb_2_R | 5.893 | 40 | 26 | -8 |
| Caudate_R | 5.865 | 14 | 14 | 14 |
| Temporal_Mid_R | 5.763 | 50 | -30 | -8 |
| Insula_R | 5.301 | 40 | -10 | -2 |

*ADHD* attention deficit hyperactivity disorder, *HC* Healthy control.

**Supplementary S1.h 10-12yr (HC) vs 10-12yr (ADHD)**

| 10-12yr (HC) vs 10-12yr (ADHD) |  | MNI Coordinates | | |
| --- | --- | --- | --- | --- |
| Region Label | t-value | x | y | z |
| Occipital_Sup_L | 5.764 | -18 | -82 | 30 |
| Cuneus_R | 5.548 | 10 | -82 | 40 |
| Precuneus_R | 4.803 | 2 | -56 | 52 |
| Occipital_Sup_R | 4.712 | 24 | -74 | 26 |

*ADHD* attention deficit hyperactivity disorder, *HC* Healthy control.
